# Severe Iron Deficiency Anemia and Growth Faltering Associated With Excessive Cow’s Milk Intake and Possible Celiac Disease in Indian Children: A Case Series

**DOI:** 10.7759/cureus.106283

**Published:** 2026-04-01

**Authors:** Anshu Mujalda, Jagdish Mujalda, Arpit Suman, Gayatri K Gupta, Manisha Manisha

**Affiliations:** 1 Obstetrics and Gynecology, Gandhi Medical College, Bhopal, IND; 2 Pediatrics, Military Hospital, Sagar, IND; 3 Preventive Medicine, Shri Ram Murti Smarak Institute of Medical Sciences (SRMS IMS), Bareilly, IND; 4 Dermatology, Skin Diseases Center, Nashik, IND; 5 Ophthalmology, SSMJ Government Hospital, Khurja, IND

**Keywords:** dietary risk factors in toddlers, excessive cow’s milk intake, iron deficiency anemia (ida), iron deficiency in indian children, pediatric nutritional anemia

## Abstract

Cow’s milk is widely considered a fundamental component of early childhood nutrition in India and frequently represents the primary daily beverage for toddlers. However, cow’s milk contains minimal bioavailable iron and may inhibit intestinal iron absorption, thereby increasing the risk of iron deficiency. Iron deficiency remains the most prevalent micronutrient deficiency in India and is the leading cause of anemia among young children. Despite national nutrition initiatives, anemia prevalence in this age group continues to be substantial, largely driven by inadequate dietary iron intake, prolonged milk-dominant feeding, suboptimal complementary feeding practices, and socioeconomic determinants.

In urban and semi-urban Indian populations, excessive cow’s milk consumption has emerged as a clinically relevant yet frequently overlooked contributor to iron deficiency in toddlers. In addition to its low iron content, high intake of cow’s milk may impair iron utilization and predispose children to occult gastrointestinal blood loss.

We describe five Indian children (age range: 48-84 months) presenting with severe iron deficiency anemia (IDA) associated with high daily intake of cow’s milk (≥600 to 1,000 mL) and inadequate consumption of iron-rich complementary foods. All patients presented with marked pallor and lethargy. Three children had poor weight gain, while two demonstrated tachycardia with clinical features suggestive of early cardiac decompensation. Hematologic investigations revealed severe microcytic hypochromic anemia with markedly reduced serum ferritin and elevated total iron-binding capacity. Three children required hospital admission, and one required pediatric intensive care unit management with packed red blood cell transfusion due to hemodynamic instability.

Management included blood transfusion when clinically indicated, therapeutic oral iron supplementation, dietary modification, and restriction of cow’s milk intake to age-appropriate levels. Follow-up at 8-12 weeks demonstrated substantial hematologic recovery in all cases.

This case series highlights the ongoing burden of nutritional IDA among Indian toddlers and underscores the importance of caregiver education, routine dietary assessment during pediatric visits, and counseling on appropriate milk intake to prevent severe anemia and its complications.

## Introduction

Iron deficiency anemia (IDA) represents one of the most prevalent nutritional disorders in early childhood, particularly among children with excessive cow’s milk consumption. Although cow’s milk is widely regarded as a dietary staple in pediatric nutrition, it contains very limited bioavailable iron. High milk intake may therefore contribute to gradual iron deficiency and subsequent anemia [1]. The prevalence of IDA among children aged 0-4 years has been reported to reach 20.1% in industrialized countries and approximately 39% in developing regions [2].

Excessive cow’s milk intake (>600 mL/day) contributes to iron deficiency by multiple mechanisms: it is inherently low in iron, its high calcium and casein content impair non-heme iron absorption, and it may induce occult intestinal blood loss. Additionally, overconsumption displaces iron-rich complementary foods, collectively increasing the risk of iron-deficiency anemia [3]. This case series illustrates the continuing burden of nutritional IDA among Indian toddlers and emphasizes the importance of caregiver awareness, early dietary evaluation during routine pediatric consultations, and appropriate counseling regarding recommended milk intake to prevent severe anemia and related complications. 

## Case presentation

Case presentations: Series of five cases

Case 1

A 5.9-year-old boy was evaluated for poor weight gain and reduced appetite. There was no history of pica, recurrent infections, gastrointestinal bleeding, helminthic infestation, chronic systemic illness, or previous blood transfusion. Developmental milestones were appropriate for age. He was born at term with a birth weight of 3 kg and did not require neonatal intensive care. Family history was negative for hemoglobinopathies. Dietary assessment revealed regular intake of more than 600 mL of cow’s milk daily since the age of two years, with minimal intake of iron-rich solid foods.

On examination, the child appeared markedly pale. Pulse rate was 110 beats/min, and respiratory rate was 22 breaths/min. There was no cyanosis, pedal edema, clubbing, or lymphadenopathy. The remainder of the systemic examination was unremarkable.

Hematological parameters: Laboratory evaluation demonstrated severe microcytic hypochromic anemia with hemoglobin 5.9 g/dL (ref value: 11-13.5 g/dL), hematocrit 24.4% (ref value: 33%-40%), and mean corpuscular hemoglobin concentration (MCHC) 24.2 g/dL (ref value: 32-36 g/dL). Red cell distribution width-coefficient of variation (RDW-CV) was elevated at 22.1% (ref value: 11.5%-14.5%), indicating significant anisocytosis. The total leukocyte count was 8.92 ×10³/µL (ref value: 5-14.5 × 10³/µL), and platelet count was 3.57 × 10⁵/µL (ref value: 1.5-4.5 × 10⁵/µL), both within normal limits. Iron studies revealed markedly reduced serum iron 23.37 µg/dL (ref value: 50-120 µg/dL) and serum ferritin 2.0 ng/mL (ref value: 12-100 ng/mL), consistent with severe iron deficiency. Vitamin D level was 11.21 ng/mL (reference range: 20-50 ng/mL), indicating deficiency. Hemoglobin electrophoresis showed HbA0 96.6% (reference range: 95%-98%), HbA2 2.4% (reference range: 2%-3.5%), and HbF 0.7% (reference range: <2%), all within normal limits. Serum IgA was normal, while tissue transglutaminase-immunoglobulin G (tTG-IgG) was positive.

Management: Packed red blood cell (PRBC) transfusion was administered at a total dose of 15 mL/kg in three divided aliquots at six-hour intervals. After hematology consultation, intravenous iron dextran therapy was initiated (100 mg daily for five days), followed by oral elemental iron supplementation.

Hospital course: The child showed progressive clinical improvement with a reduction in pallor and improved activity. No transfusion-related complications occurred. Stool guaiac testing was positive for occult blood, suggesting intestinal inflammation related to excessive cow’s milk intake.

Discharge advice summary: The child was discharged in stable clinical condition. Parents received counseling regarding restriction of excessive cow’s milk intake and introduction of iron-rich foods with improved dietary diversity. Oral elemental iron was continued at 3-6 mg/kg/day in divided doses. At one-month follow-up, the child demonstrated improved activity, appetite, and significant recovery of hemoglobin levels.

Case 2

A 6.3-year-old boy presented with progressive fatigue, decreased physical activity, and poor appetite over three months. There was no history of gastrointestinal bleeding, parasitic infestation, chronic illness, or previous blood transfusion. Birth and developmental histories were normal. Dietary history revealed consumption of approximately 800 mL of cow’s milk daily with poor intake of iron-rich foods.

Physical examination revealed severe pallor with mild tachycardia (pulse rate 118 beats/min). Respiratory rate was 24 breaths/min. No hepatosplenomegaly, lymphadenopathy, or cardiac failure was noted.

Hematological parameters: Laboratory investigations showed hemoglobin 6.3 g/dL (ref value: 11-13.5 g/dL), hematocrit 26.1% (ref value: 33%-40%), and MCHC 25.0 g/dL (ref value: 32-36 g/dL), consistent with hypochromic anemia. RDW-CV was elevated at 21.4% (ref value: 11.5%-14.5%). The TLC was 9.10 × 10³/µL (ref value: 5-14.5 × 10³/µL) and platelet count 3.80 × 10⁵/µL (ref value: 1.5-4.5 × 10⁵/µL), both within normal limits. Iron studies revealed serum iron 28.10 µg/dL (ref value: 50-120 µg/dL) and serum ferritin 3.1 ng/mL (ref value: 12-100 ng/mL), indicating depleted iron stores. Vitamin D level was 14.30 ng/mL (ref value: 20-50 ng/mL), consistent with deficiency. Hemoglobin electrophoresis demonstrated HbA0 96.1% (ref value: 95%-98%), HbA2 2.6% (ref value: 2%-3.5%), and HbF 0.9% (ref value: <2%), all within normal limits. Serum IgA was normal, and tTG-IgG was negative.

Management: PRBC transfusion (15 mL/kg) was administered in three divided aliquots at six-hour intervals. Intravenous iron dextran therapy (100 mg daily for five days) was initiated, followed by oral elemental iron supplementation.

Hospital course: The patient tolerated transfusion and iron therapy well, with progressive improvement in activity and appetite during hospitalization. Stool occult blood testing was positive and considered consistent with cow’s milk-associated intestinal inflammation.

Discharge advice summary: The child was discharged in stable condition with dietary counseling emphasizing restriction of excessive cow’s milk intake and increased consumption of iron-rich foods. Oral iron therapy was continued at 3-6 mg/kg/day. At one-month follow-up, clinical and hematologic parameters showed significant improvement.

Case 3

A 5.6-year-old girl presented with easy fatigability, reduced scholastic attention, and inadequate weight gain. There was no history suggestive of pica, parasitic infection, bleeding disorder, or chronic illness. Birth history and development were normal. Dietary assessment revealed intake of approximately 700 mL of cow’s milk daily with limited consumption of iron-rich foods.

Examination revealed pallor with pulse rate 112 beats/min and respiratory rate 20 breaths/min. Systemic examination was otherwise normal.

Hematological parameters: Laboratory findings revealed hemoglobin 5.6 g/dL (ref value: 11-13.5 g/dL), hematocrit 23.8% (ref value: 33-40%), and MCHC 23.9 g/dL (ref value: 32-36 g/dL), consistent with severe microcytic hypochromic anemia. RDW-CV was markedly elevated at 23.0% (ref value: 11.5%-14.5%). The TLC was 8.45 ×10³/µL (ref value: 5-14.5 × 10³/µL), and platelet count 3.40 × 10⁵/µL (ref value: 1.5-4.5 × 10⁵/µL). Iron parameters showed serum iron 19.8 µg/dL (ref value: 50-120 µg/dL) and serum ferritin 1.8 ng/mL (ref value: 12-100 ng/mL), confirming severe iron deficiency. Vitamin D level was 10.8 ng/mL (ref value: 20-50 ng/mL), indicating deficiency. Hemoglobin electrophoresis revealed HbA0 97.0% (ref value: 95%-98%), HbA2 2.3% (ref value: 2%-3.5%), and HbF 0.6% (ref value: <2%), within normal limits. Serum IgA was normal, and tTG-IgG was positive.

Management: The child received a PRBC transfusion (15 mL/kg) in divided aliquots at six-hour intervals. Intravenous iron dextran (100 mg daily for five days) was administered, followed by oral elemental iron therapy.

Hospital course: Clinical improvement was observed during hospitalization with increased activity and improved appetite. No adverse transfusion reactions occurred. Laboratory findings supported the diagnosis of severe iron deficiency anemia.

Discharge advice summary: The patient was discharged in stable condition with advice to limit excessive cow’s milk intake and increase dietary iron sources. Oral iron therapy was continued at 3-6 mg/kg/day. At follow-up after one month, the child demonstrated improved hemoglobin levels and better nutritional intake.

Case 4

A 6.1-year-old boy presented with lethargy, irritability, and poor appetite for two months. There was no history of gastrointestinal bleeding, parasitic infection, chronic illness, or previous hospitalization. Dietary evaluation revealed excessive cow’s milk intake exceeding 900 mL daily, with poor intake of solid foods.

Clinical examination demonstrated marked pallor and tachycardia (pulse rate 122 beats/min). Respiratory rate was 26 breaths/min. No organomegaly or lymphadenopathy was present.

Hematological parameters: Laboratory parameters showed hemoglobin 6.1 g/dL (ref value: 11-13.5 g/dL), hematocrit 25.2% (ref value: 33%-40%), and MCHC 24.8 g/dL (ref value: 32-36 g/dL). RDW-CV was 22.6% (ref value: 11.5%-14.5%), indicating anisocytosis. The TLC was 9.30 × 10³/µL (ref value: 5-14.5 × 10³/µL), and platelet count 3.65 × 10⁵/µL (ref value: 1.5-4.5 × 10⁵/µL). Iron profile revealed serum iron 25.40 µg/dL (ref value: 50-120 µg/dL) and serum ferritin 2.5 ng/mL (ref value: 12-100 ng/mL), consistent with iron deficiency anemia. Vitamin D level was 13.50 ng/mL (ref value: 20-50 ng/mL), indicating deficiency. Hemoglobin electrophoresis demonstrated HbA0 95.8% (ref value: 95%-98%), HbA2 2.5% (ref value: 2%-3.5%), and HbF 1.0% (ref value: <2%), all within normal limits. Serum IgA was normal, and tTG-IgG was negative.

Management: PRBC transfusion (15 mL/kg) was administered in three divided doses at six-hour intervals. Intravenous iron dextran (100 mg daily for five days) was given, followed by oral elemental iron supplementation.

Hospital course: The patient showed steady clinical improvement during admission without transfusion-related complications. Positive stool occult blood testing suggested intestinal mucosal irritation associated with excessive cow’s milk intake.

Discharge advice summary: The child was discharged with counseling regarding the reduction of milk intake and improvement of dietary diversity. Oral elemental iron therapy was continued at 3-6 mg/kg/day. At one-month follow-up, clinical and hematologic improvement was documented.

Case 5

A 5.8-year-old girl presented with poor feeding, decreased play activity, and inadequate weight gain. There was no history of recurrent infections, bleeding disorders, worm infestation, or chronic illness. Birth and developmental histories were unremarkable. Dietary history revealed intake of approximately 750 mL of cow’s milk daily with inadequate introduction of iron-rich foods.

On examination, she appeared markedly pale. Pulse rate was 120 beats/min, and respiratory rate was 24 breaths/min. No hepatosplenomegaly or systemic abnormalities were noted.

Hematological parameters: Laboratory investigations revealed hemoglobin 5.8 g/dL (ref value: 11-13.5 g/dL), hematocrit 24.0% (ref value: 33%-40%), and MCHC 24.5 g/dL (ref value: 32-36 g/dL). RDW-CV was elevated at 21.9% (ref value: 11.5%-14.5%). The TLC was 8.70 × 10³/µL (ref value: 5-14.5 × 10³/µL) and platelet count 3.50 × 10⁵/µL (ref value: 1.5-4.5 × 10⁵/µL). Iron studies showed serum iron 21.60 µg/dL (ref value: 50-120 µg/dL) and serum ferritin 2.2 ng/mL (ref value: 12-100 ng/mL), confirming severe iron deficiency. Vitamin D level was 12.60 ng/mL (ref value: 20-50 ng/mL), indicating deficiency. Hemoglobin electrophoresis revealed HbA0 96.4% (ref value: 95%-98%), HbA2 2.2% (ref value: 2%-3.5%), and HbF 0.8% (ref value: <2%), within normal limits. Serum IgA was normal, while tTG-IgG was borderline positive.

Management: PRBC transfusion was administered at a dose of 15 mL/kg in three divided aliquots at six-hour intervals. Intravenous iron dextran therapy (100 mg daily for five days) was initiated, followed by oral iron supplementation.

Hospital course: The patient showed gradual clinical improvement with increased activity and improved appetite. No transfusion-related complications were noted. Evaluation confirmed severe iron deficiency anemia associated with excessive cow’s milk intake and poor dietary iron intake.

Discharge advice summary: The patient was discharged in stable clinical condition with counseling regarding the limitation of cow’s milk intake and improvement of dietary diversity. Oral iron therapy was continued at 3-6 mg/kg/day. At one-month follow-up, hemoglobin levels improved significantly with recovery from iron deficiency anemia.

Follow-up

At one-month follow-up, all children demonstrated significant clinical improvement with increased activity levels and better appetite. Hemoglobin levels improved substantially following iron therapy and dietary modification. Caregivers were advised to continue oral elemental iron at a therapeutic dose of 3-6 mg/kg/day in divided doses, administer iron between meals when feasible, and combine it with vitamin C-containing foods to enhance absorption. Dietary counseling emphasized limiting cow’s milk intake and increasing consumption of iron-rich foods to prevent recurrence of iron deficiency anemia.

The demographic characteristics of all five cases are summarized in Table 1.

**Table 1:** Demographical characteristics of all five children.

Characteristics	Case 1	Case 2	Case 3	Case 4	Case 5
Age ( in years)	5.9	6.3	5.6	6.1	5.8
Sex	M	M	F	M	F
Weight (in kg)	16.8	15.2	14.6	15.4	15.1
Height (in cm)	108	110	102	99	103
Daily cow milk intake (in 24 hours)	>600 mL	>600 mL	>600 mL	>600 mL	>600 mL

Laboratory Findings

The comparative laboratory parameters of the five children are presented in Table 2. All patients demonstrated reduced hemoglobin, hematocrit, MCHC, serum iron, and ferritin levels, consistent with iron deficiency anemia. RDW values were elevated in all cases, whereas total leukocyte and platelet counts remained within normal limits. Hemoglobin fraction analysis (HbA0, HbA2, and HbF) did not reveal significant abnormalities. Vitamin D levels were reduced in all patients. tTG-IgG positivity was identified in two children, with one additional case showing borderline positivity.

**Table 2:** Laboratory parameters - comparative summary of all five cases. *Kliegman et al. [4]. Hb, hemoglobin; HbA0, adult hemoglobin A0; HbA2, adult hemoglobin A2; HbF, fetal hemoglobin; Hct, hematocrit/packed cell volume; MCHC, mean corpuscular hemoglobin concentration; RDW-CV, red cell distribution width-coefficient of variation; TLC, total leukocyte count; IgA, immunoglobulin A; tTG-IgG, tissue transglutaminase-immunoglobulin G

Test	Ref value*	Case 1	Case 2	Case 3	Case 4	Case 5
Hemoglobin (g/dL)	11.0-13.5	5.9	6.3	5.6	6.1	5.8
Hematocrit (%)	33-40	24.4	26.1	23.8	25.2	24.0
MCHC (g/dL)	32-36	24.2	25.0	23.9	24.8	24.5
RDW-CV (%)	11.5-14.5	22.1	21.4	23.0	22.6	21.9
TLC (×10³/µL)	5.0-14.5	8.92	9.10	8.45	9.30	8.70
Platelets (×10⁵/µL)	1.5-4.5	3.57	3.80	3.40	3.65	3.50
Serum iron (µg/dL)	50-120	23.37	28.10	19.80	25.40	21.60
Serum ferritin (ng/mL)	12-100	2.0	3.1	1.8	2.5	2.2
Vitamin D (ng/mL)	20-50	11.21	14.30	10.80	13.50	12.60
HbA0 (%)	95-98	96.6	96.1	97.0	95.8	96.4
HbA2 (%)	2.0-3.5	2.4	2.6	2.3	2.5	2.2
HbF (%)	<1-2% after infancy	0.7	0.9	0.6	1.0	0.8
Serum IgA	20-200	90	102	135	95	83
tTG-IgG	<15 U/mL - negative	Positive	Negative	Positive	Negative	Borderline positive

Overall Management

All children required admission to the pediatric intensive care unit (PICU). Evaluation suggested that the anemia was secondary to iron deficiency resulting from inadequate dietary iron intake combined with chronic occult gastrointestinal blood loss associated with cow’s milk protein-induced colitis. Clinical features included marked pallor, fatigue, and decreased activity levels. Laboratory investigations demonstrated microcytic hypochromic anemia, and stool guaiac testing was positive for occult blood in all cases. A detailed dietary assessment confirmed insufficient iron intake with excessive cow’s milk consumption.

All children received PRBC transfusion at a total dose of 15 mL/kg, administered as three divided aliquots at six-hour intervals. Following hematology consultation, intravenous iron dextran therapy was initiated (100 mg daily for five days), followed by maintenance oral elemental iron supplementation (30 mg daily).

Overall Hospital Course

During hospitalization, all children demonstrated progressive clinical improvement, with increased activity levels and a gradual reduction in pallor. No transfusion-related complications were observed. The presence of occult blood in stool was attributed to inflammatory colitis secondary to cow’s milk protein exposure. Hematology evaluation confirmed the diagnosis of severe iron deficiency anemia in all cases, based on clinical findings, dietary history, laboratory investigations, and positive stool guaiac testing.

Discharge and Follow-Up

All children were discharged in stable clinical condition following appropriate management. Parents and caregivers received detailed dietary counseling emphasizing the limitation of cow’s milk intake, introduction of iron-rich foods, and diversification of the child’s diet to ensure adequate nutritional intake. Referral for outpatient nutritional counseling was arranged for continued dietary monitoring and reinforcement of feeding practices.

Oral iron therapy was continued at a therapeutic dose of 3-6 mg/kg/day of elemental iron in divided doses, as recommended for the treatment of pediatric iron deficiency anemia. Caregivers were advised to administer iron between meals when possible and with vitamin C-containing foods to enhance absorption.

At one-month follow-up, all children demonstrated clinical improvement with increased activity levels and improved appetite. Hemoglobin levels showed significant recovery following iron supplementation and dietary modification. The children resumed regular intake of solid foods, and follow-up laboratory parameters indicated satisfactory hematologic improvement consistent with recovery from iron deficiency anemia.

## Discussion

Iron deficiency and IDA remain important public health concerns among toddlers, with excessive cow’s milk intake recognized as a significant and modifiable risk factor. During the transition from infancy to early childhood, adherence to appropriate feeding practices is essential to maintain adequate iron stores. Failure to implement preventive dietary strategies may predispose children to clinically significant anemia and potential neurodevelopmental impairment.

The relationship between excessive cow’s milk intake and iron deficiency is multifactorial and well described. Cow’s milk contains very low iron concentrations (approximately 0.5 mg/L), comparable to goat’s and sheep’s milk, while mare’s milk contains only slightly higher amounts, as reported by Pietrzak-Fiećko et al. [5]. High milk intake often displaces iron-rich complementary foods, leading to reduced dietary iron consumption and poor micronutrient density.

**Figure 1:**
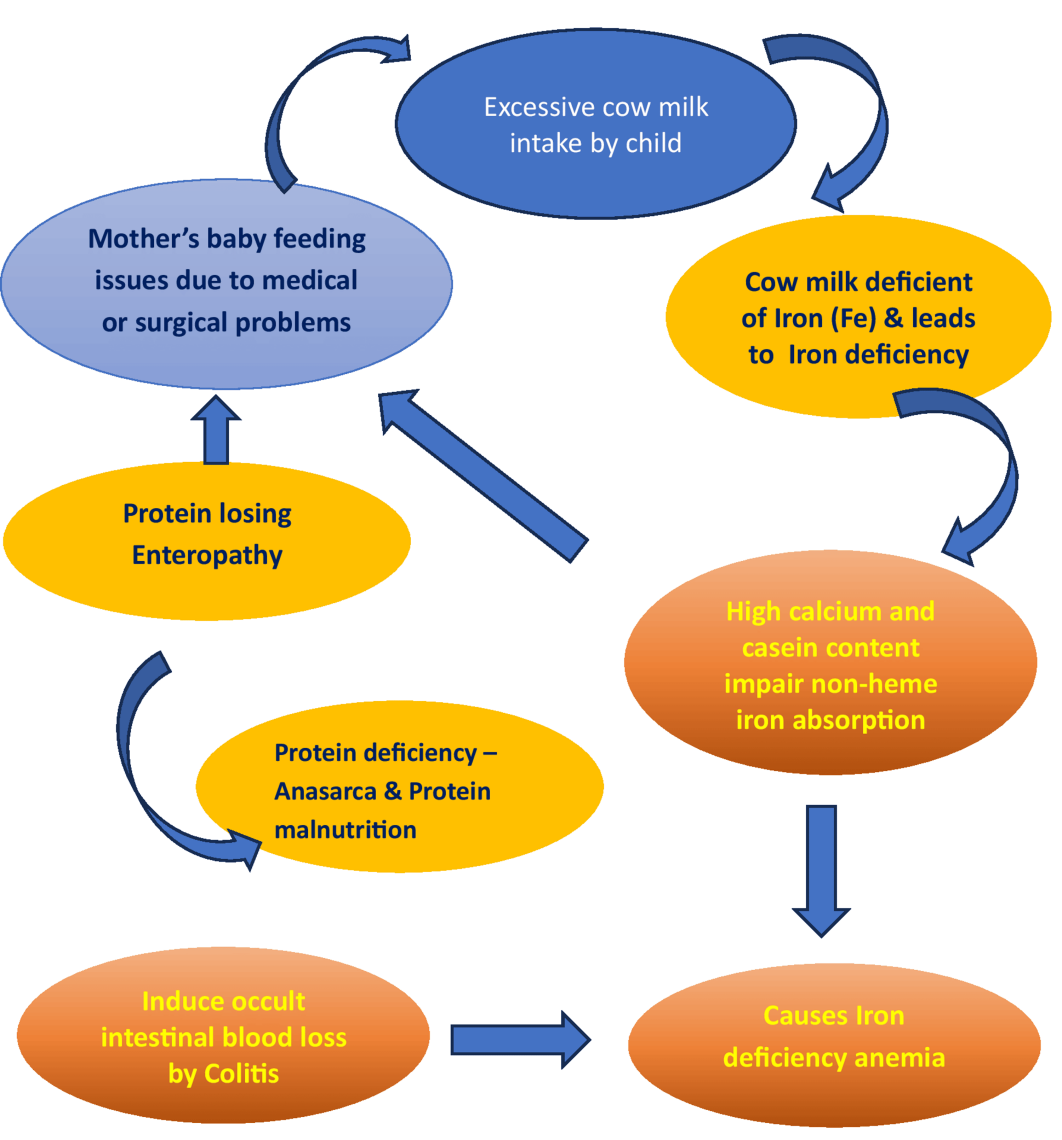
Pathophysiological cascade of excessive cow’s milk intake leading to iron deficiency anemia. Cow’s milk contains approximately 0.03-0.05 mg of iron per 100 mL, and casein constitutes ~80% of total milk protein; its high calcium (~120 mg/100 mL) and casein content inhibit non-heme iron absorption and also induce colitis, which also contributes to occult blood loss in susceptible children. Image credit: All authors.

IDA is commonly reported among children from lower socioeconomic groups in developed countries. However, the children described in this case series belonged to economically stable families. In these cases, anemia was primarily associated with excessive intake of unfortified pasteurized cow’s milk combined with inadequate consumption of iron-rich foods. Progressive iron deficiency may further suppress appetite, worsening nutritional intake and perpetuating a cycle of anorexia and iron depletion that can ultimately result in severe complications (Figure 1).

In addition to its low intrinsic iron content, cow’s milk negatively influences iron bioavailability. The iron present in cow’s milk is predominantly in the non-heme form, which is absorbed less efficiently than heme iron Levy-Costa et al. [6]. Furthermore, the high concentrations of calcium and casein in cow’s milk interfere with intestinal iron absorption, particularly non-heme iron, which represents the major form of dietary iron in early childhood diets Levy-Costa et al. [6]. Cow’s milk contains approximately four times more calcium than human milk, and this increased calcium load competes with iron for absorption at the level of enterocytes Vanderhoof et al. [7].

Another contributing factor is the low vitamin C content of cow’s milk, which may be further reduced during pasteurization Vanderhoof et al. [7]. Vitamin C facilitates the absorption of non-heme iron by reducing ferric iron to the more absorbable ferrous form; therefore, reduced vitamin C availability further compromises intestinal iron uptake. Although the absolute iron concentrations of cow’s milk and human milk are comparable, the bioavailability of iron from human milk is approximately 2.5-fold higher Bondi et al. [8]. This difference highlights the increased vulnerability to iron deficiency following the early transition from breast milk to unfortified cow’s milk.

Occult gastrointestinal blood loss represents an additional mechanism contributing to iron depletion. Small amounts of physiological gastrointestinal blood loss occur in healthy infants; however, excessive cow’s milk consumption has been associated with increased occult bleeding in susceptible children, thereby increasing iron requirements Wilson et al. [9]. Children with concurrent protein-losing enteropathy and IDA demonstrate higher fecal hemoglobin concentrations (approximately 1.48 mg/g of stool) compared with reference values of 0.5-0.8 mg/g Salstrom et al. [10]. Nevertheless, gastrointestinal bleeding alone does not appear sufficient to explain severe IDA in most cases. Evidence suggests that anemia develops through a combined effect of inadequate intake, impaired absorption, and increased iron loss Lundstrom et al. [11].

Recent investigations continue to demonstrate an inverse relationship between iron status and cow’s milk intake during infancy and early childhood Gul et al. [12]. Excessive milk consumption, therefore, remains a significant and modifiable risk factor for iron deficiency, consistent with the public health evaluation reported by Chouraqui et al. [13]. Maintenance of normal iron homeostasis requires adequate dietary intake, efficient intestinal absorption, and minimal physiological loss; excessive cow’s milk intake adversely affects all three mechanisms and substantially increases the risk of IDA.

Children whose diets consist predominantly of cow’s milk have been shown to have a higher prevalence of IDA compared with those receiving breast milk or iron-fortified formula Gul et al. [12]. Because cow’s milk provides considerable caloric intake with relatively low iron density, it may promote satiety while simultaneously displacing iron-rich foods from the diet, thereby compounding the risk of deficiency Chouraqui et al. [13]. The inhibitory effects of calcium and casein on non-heme iron absorption, combined with the low vitamin C content of cow’s milk, further amplify this risk.

Graczykowska et al. [14] emphasized that occult gastrointestinal bleeding may further aggravate iron depletion in susceptible individuals. Similarly, the potential severity of this dietary pattern has been illustrated in recent literature. Nelson et al. [15] reported a case of a 2-year-old child presenting with profound anemia (hemoglobin 2.0 g/dL) attributed to excessive cow’s milk consumption, highlighting the possibility of life-threatening complications.

Although moderate intake of cow’s milk can be included as part of a balanced pediatric diet, excessive consumption is consistently associated with an increased risk of iron deficiency, as noted by Wong [16]. The American Academy of Pediatrics recommends limiting cow’s milk intake to approximately 16-24 ounces (480-720 mL) per day in children aged 1-5 years to reduce the likelihood of nutritional deficiencies, including iron deficiency DiMaggio [17].

Current pediatric nutritional guidelines, therefore, emphasize restricting excessive milk consumption, encouraging dietary diversification with iron-rich complementary foods, and implementing age-appropriate screening strategies to prevent adverse neurodevelopmental outcomes associated with early iron deficiency Coppola et al. [18].

In summary, excessive cow’s milk intake contributes to iron deficiency in toddlers through three interrelated mechanisms: inadequate dietary iron density, impaired intestinal absorption, and potential gastrointestinal blood loss. Given that this risk factor is largely preventable, excessive milk intake should remain an important target for anticipatory guidance, dietary counseling, and public health interventions aimed at reducing the burden of iron deficiency and IDA during early childhood.

## Conclusions

Severe iron deficiency anemia in children has also been associated with hypoalbuminemia, particularly in the context of excessive cow’s milk consumption. Although the exact pathophysiological mechanism remains incompletely understood, this relationship appears to occur independently of cow’s milk protein allergy (CMPA). In pediatric patients presenting with edema in the presence of iron deficiency anemia, clinicians should consider protein-losing enteropathy related to excessive cow’s milk intake in the differential diagnosis.

Adequate nutritional balance is essential during early childhood. Cow’s milk should not function as the primary dietary source, and pediatric diets must include sufficient iron to support normal growth and neurodevelopment. Routine pediatric evaluations should therefore incorporate careful dietary assessment during the early years of life. Increased awareness among pediatricians is necessary regarding the potential association between profound iron deficiency anemia and generalized edema, particularly given the widespread reliance on milk in young children’s diets and the relative rarity of this presentation. Further research is warranted to clarify the role of iron in maintaining intestinal barrier integrity and regulating protein dynamics within the gastrointestinal tract.
